# Tonoplast sugar transporters as key drivers of sugar accumulation, a case study in sugarcane

**DOI:** 10.1093/hr/uhae312

**Published:** 2024-11-06

**Authors:** Michael Tang, Jiang Wang, Baskaran Kannan, Niki Maria Koukoulidis, Yi-Hsuan Lin, Fredy Altpeter, Li-Qing Chen

**Affiliations:** School of Integrative Biology, College of Liberal Arts & Sciences, University of Illinois Urbana-Champaign, Urbana, IL 61801, United States; Department of Plant Biology, University of Illinois Urbana-Champaign, Urbana, IL 61801, United States; Department of Energy (DOE) Center for Advanced Bioenergy and Bioproducts Innovation, University of Illinois Urbana-Champaign, Urbana, IL 61801, United States; Agronomy Department, Plant Molecular and Cellular Biology Program, Genetics Institute, University of Florida, IFAS, Gainesville, FL 32603, United States; DOE Center for Advanced Bioenergy and Bioproducts Innovation, Gainesville, FL 32603, United States; Agronomy Department, Plant Molecular and Cellular Biology Program, Genetics Institute, University of Florida, IFAS, Gainesville, FL 32603, United States; DOE Center for Advanced Bioenergy and Bioproducts Innovation, Gainesville, FL 32603, United States; Department of Plant Biology, University of Illinois Urbana-Champaign, Urbana, IL 61801, United States; Department of Energy (DOE) Center for Advanced Bioenergy and Bioproducts Innovation, University of Illinois Urbana-Champaign, Urbana, IL 61801, United States; Agronomy Department, Plant Molecular and Cellular Biology Program, Genetics Institute, University of Florida, IFAS, Gainesville, FL 32603, United States; DOE Center for Advanced Bioenergy and Bioproducts Innovation, Gainesville, FL 32603, United States; Department of Plant Biology, University of Illinois Urbana-Champaign, Urbana, IL 61801, United States; Department of Energy (DOE) Center for Advanced Bioenergy and Bioproducts Innovation, University of Illinois Urbana-Champaign, Urbana, IL 61801, United States

##  

Dear Editor,

Accumulation of sugars, such as glucose, fructose, sucrose, and sorbitol, is a very important trait in crops. It significantly impacts the yield of sugar crops as well as fruit quality, taste, and marketability of horticultural crops [1–3]. Sugarcane not only provides 80% of the table sugar but also 40% of the biofuel produced globally [4]. In addition, sugarcane is also an important model for studying sugar accumulation in crops. In mature sugarcane stems, the sucrose concentrations range from 400 to 700 mM, accounting for up to 30% of plant fresh weight [4]. However, the mechanism underlying high sugar accumulation in sugarcane, as well as in many horticulture crops, remains relatively underexplored.

Tonoplast sugar transporters (TSTs) are known to play a critical role in transporting sugars into vacuoles for sugar accumulation, such as *CmTST2* in watermelon, *CsTST1* and *CsTST2* in cucumber, and *MdTST1* and *MdTST2* in apple [3]. Tonoplast monosaccharide transporters (TMTs) were first reported in *Arabidopsis* to mediate glucose and fructose transport in the tonoplast, especially in cold-adapted plants. In 2015, TMTs were renamed as TSTs due to the sucrose transport specificity of highly expressed *BvTST2.1* in sugar beet [5]. The biochemical understanding of TSTs was advanced by electrophysiological studies of BvTST2.1, which operates as a proton antiporter, coupling sucrose import into the vacuole with proton export [5].

Given the significance of TSTs in sugar accumulation in other species, it is reasonable to predict that TSTs might also play an important role in sugar accumulation in sugarcane. The highly polyploid nature of the sugarcane genome, however, presents great challenges to identifying which *TST*s are major contributors to sugar accumulation. A few years ago, a mosaic monoploid sugarcane genome sequence was published [6], enabling to manipulate *TST* expression in sugarcane.

In 2021, our group identified stem-specific/preferred *TST* promoters in sugarcane [7]. Modern sugarcane cultivars are allopolyploid hybrids of *Saccharum officinarum* (sugary ancestor) and *Saccharum spontaneum* (resilient ancestor) and typically have 10–13 homologous alleles per locus. Using an allele-defined genome of autopolyploid sugarcane via a haploid version of *S. spontaneum* and an RNA-seq atlas of *S. spontaneum*, we identified five TST genes with 21 alleles in the *S. spontaneum* genome. Through qPCR, semi-RT–PCR, RNA *in situ*, and promoter-GUS analysis, we determined that *TST2b-1A and TST2b-1C* are strongly and nearly exclusively expressed in sugarcane stem, while *TST1* is preferentially expressed in the stem [7]. Their expression specificity, particularly in pith parenchyma cells of the stem, makes them ideal for understanding sugar accumulation, given that sugarcane stems comprise 70% of the plant’s biomass at harvest.

Based on our previous findings and other reported results, we predicted that TST1 and TST2b-1A and TST2b-1C are responsible for sugar accumulation in the vacuoles of sugarcane stems. To test this hypothesis, we generated simultaneous knockdown events for these genes using the RNA interference (RNAi) strategy in sugarcane cultivar UFCP84–1047 (Fig. 1a), a high-fiber sugarcane cultivar for cellulosic ethanol production. For this purpose, we employed particle bombardment-mediated transformation. Following biolistic gene transfer and selection with a geneticin-containing culture medium [8], a total of eight independent transgenic plants harboring the TST-RNAi construct were generated and confirmed by PCR analysis. Due to the high sequence similarity between allele *TST2b-1A* and allele *TST2b-1C* and given that *TST2b-1A* contributes significantly to the total expression of *TST2b* [7], we used our primers, designed as described by Wang *et al*. publication [7], targeting conserved regions of both *TST2b-1A* and *TST2b-1C*. Additionally, due to the overall high sequence similarity among TST members, we also evaluated the effects of RNAi on the expression of *TST2a*, which is also highly and preferentially expressed in the stem, while the expression levels of *TST3a* and *TST3b* are under the reliable detection range (data not shown), consistent with our previous findings [7]. We selected four sugarcane events that exhibited a >2-fold reduction in the expression levels of all tested genes by quantitative PCR (qPCR) from the tissue of internode 5 (counting from the top of the plant) of 7-month-old sugarcane plants (Fig. 1b). These RNAi events did not show significant phenotypic differences from the transformation control (TC) at maturity under our greenhouse conditions, including height (Fig. 1c) and diameter (Fig. 1d). Additionally, as predicted, all four RNAi lines showed a statistically significant reduction in total soluble sugars of internode 5 compared with the TC (Fig. 1e). The total sugar level of RNAi lines was, on average, reduced by 80% relative to the total sugar levels in the TC.

**Figure 1:**
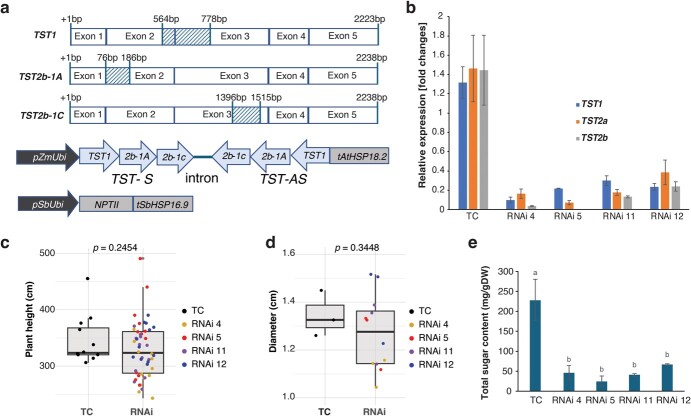
**a** Schematic representation of RNA interference target regions in *TST1*, *TST2b-1A*, and *TST2b-1C* marked with diagonal hatching. *TST-RNAi* and *NPTII* are marker gene constructs used for biolistic transformation. *TST-S*, sense strands of *TST1*, *TST2b-1A*, and *TST2b-1C*; *TST-AS*, anti-sense strands *TST1*, *TST2b-1A*, and *TST2b-1C*; intron, *4CL* intron from bahiagrass. **b** Relative gene expression levels of *TST1*, *TST2a*, and *TST2b* in internode 5 were determined by qPCR. *GAPDH* (glyceraldehyde 3-phosphate dehydrogenase) served as an internal control for normalization at maturity of plants (mean ± standard error, *n* = 3). **c** Height of stems from 7-month-old plants showed no significant differences between the TC and RNAi lines (*n* = 12, each background). **d** Diameter of internode 15 of TC and RNAi events at 7 months old (*n* = 3, each background). The boxes are drawn from the 25th percentile to the 75th percentile and the medians are shown. Whiskers extend to 1.5 times the interquartile range from the 25th and 75th percentiles. Student’s *t*-test was used for the significance test. Plants were grown under controlled temperature (28°C/22°C, day/night) with a 14-h light (~300–950 μmol m^−2^ s^−1^)/10-h dark regimen via a mix of natural photoperiod and light conditions. **e** Total soluble sugar (sucrose + glucose + fructose) contents using high-performance liquid chromatography (HPLC) equipped with a refractive index detector (RID) (mean ± standard error, *n* = 3). Statistically significant differences among samples were determined using one-way ANOVA followed by multiple comparison tests (Fisher’s LSD method) with different letters indicating significant differences (*P* < 0.05).

Our data strongly demonstrate that *TST1*, *TST2a*, and *TST2b* significantly contribute to sugar accumulation in sugarcane, supporting the conserved function of TSTs in sugar accumulation across various species. The absence of obvious phenotypic differences between the TC and RNAi lines may be attributed to several factors. Residual levels of *TST1*, *TST2a*, and *TST2b* might be sufficient to meet the growth needs of plants under our greenhouse conditions. Sugarcane undergoes significant sugar accumulation during the maturation phase after the grand growth phase, during which it reaches its peak growth and fully develops the canopy [9]. So, growth and sugar accumulation predominantly occur at different stages, with TSTs playing a crucial role in sugar accumulation. In addition, sugar accumulation enhancement in sugarcane largely results from breeding selection and domestication efforts [4]. Prior to selection for high sugar content, growth may have only required baseline levels of *TST* expression, which requires further investigation. Knockout mutants, such as those generated via CRISPR editing, would be valuable for addressing this question. Furthermore, functional redundancy among TST members could compensate for the suppression of specific genes. Other tonoplast-localized sugar transporters, such as tonoplast-localized SWEETs (sugars will eventually be exported transporters) and SUTs (sucrose transporters) [3], may also play compensatory roles in sugar transport.

While we did not observe significant phenotypes under our greenhouse conditions, field evaluation will be necessary for the final conclusion regarding the agronomic performance of these RNAi plants. For example, *zmsut1, a* phloem loading-impaired mutant of maize, showed strong phenotypic differences relative to control plants under summer field conditions but not under winter greenhouse conditions [10].

## Data Availability

The data underlying this article will be shared on reasonable request to the corresponding author.
